# The Effects of Different Insufflation Pressures on Liver Functions Assessed with LiMON on Patients Undergoing Laparoscopic Cholecystectomy

**DOI:** 10.1100/2012/172575

**Published:** 2012-04-24

**Authors:** H. Barıs Eryılmaz, Dilek Memiş, Atakan Sezer, Mehmet Turan Inal

**Affiliations:** ^1^Department of Anaesthesiology and Reanimation, Trakya University Medical Faculty, 22030 Edirne, Turkey; ^2^Department of Surgery, Trakya University Medical Faculty, 22030 Edirne, Turkey

## Abstract

*Purpose*. Laparoscopic cholecystectomy has been accepted as an alternative to laparotomy, but there is still controversy regarding the effects of pneumoperitoneum on splanchnic and hepatic perfusion. We assessed the effects of different insufflation pressures on liver functions by using indocyanine green elimination tests (ICG-PDR). *Methods*. We analyzed 43 patients who were scheduled for laparoscopic cholecystectomy. The patients were randomly allocated to two groups. In Group I, the operation was performed using 10 mmHg pressure pneumoperitoneum. In Group II, 14 mmHg pressure pneumoperitoneum was used. The ICG-PDR measurements were made after induction (ICG-PDR 1) and after the end of the operation (ICG-PDR 2). Serum aspartate aminotransferase (AST), alanine aminotransferase (ALT), and total bilirubin levels were all recorded preoperatively, 1 hour, and postoperative 24 hours after surgery. *Results*. The ICG-PDR 1 values for Groups I and II were as follows: 26.78 ± 4.2% per min versus 26.01 ± 2.4% per min (*P* > 0.05). ICG-PDR 2 values were found to be 25.63 ± 2.1% per min in Group I versus 19.06 ± 2.2% per min in Group II (*P* < 0.05). There was a statistically significant decrease between baseline and postoperative ICG-PDR values in Group II compared to Group I (*P* < 0.05). Statistically, there was an increase between baseline and postoperative 1st-hour serum AST and ALT level in Group II (*P* < 0.05) compared to Group I. No statistical differences were detected on postoperative 24st-hour serum AST and ALT levels and all the time bilirubin between groups (*P* > 0.05). *Conclusion*. In conclusion, the results show that 14 mmHg pressure pneumoperitoneum decreased the blood flow to the liver and increased postoperative 1st-hour serum AST and ALT levels. We think that 10 mmHg pressure pneumoperitoneum is superior to 14 mmHg pressure pneumoperitoneum in laparoscopic cholecystectomy.

## 1. Introduction

Laparoscopic cholecystectomy is widely used for treatment of benign gallbladder diseases. Today more than 90% of cholecystectomies are performed laparoscopically [1–3].

A transient increase in intra-abdominal pressure (IAP), such as pneumoperitoneum during laparoscopic surgery, causes only minimal adverse effects [4–6]. Several studies have demonstrated that the serum level of liver enzymes rises markedly after laparoscopic surgeries, and this increase may be attributable to impaired liver and splanchnic perfusion [7–11]. Hasukic [12] demonstrated that these liver enzymes increased more after high-pressure pneumoperitoneum than after low-pressure pneumoperitoneum. 

Plasma disappearance rate of indocyanine green (ICG-PDR) is a dynamic test for the assessment of liver function, and this has been suggested as a marker of global hepatosplanchnic blood flow [13, 14]. The ICG-PDR can be assessed with a noninvasive liver function monitoring system, the LiMON (Pulsion Medical Systems, Munich, Germany) [15, 16]. Our recent study [17] found that the measurement of ICG-PDR with a LiMON is a good predictor for determining the effects of IAP on liver functions.

The aim of the present study was to examine the effects of different insufflation pressures on liver function as assessed with LiMON on patients scheduled for laparoscopic cholecystectomy.

## 2. Materials and Methods

The Regional Committee on Medical Research Ethics approved the study. Informed, written consent was obtained from each patient. Forty-three ASA physical status I or II patients undergoing elective laparoscopic cholecystectomy were included in the study. Patients with liver failure, coagulopathy, and known allergy to medication drugs were excluded from the study.

The same senior surgeon (A. Sezer) operated all patients, and a similar anesthesia protocol was administered to all patients. Midazolam hydrochloride, used for premedication, was given 45 minutes before surgery at a dose of 0.7 mg/kg. Following 2 minutes of preoxygenation, anesthesia was induced using fentanyl citrate (1–1.5 *μ*g/kg), propofol (2–2.5 mg/kg), as well as vecuronium (0.1 mg/kg) for muscle relaxation. Anesthesia was maintained with sevoflurane (2–2.5 vol%) and oxygen-in-air mixture (0.50 ratio). Ventilation was mechanically controlled to maintain normocapnia (end-tidal carbon dioxide, 35–38 mmHg).

The patients were randomly allocated to two groups. In Group I, the operation was performed using 10 mmHg pressure pneumoperitoneum. In Group II, 14 mmHg pressure pneumoperitoneum was used.

Haemodynamic variables (heart rate, mean arterial pressure, peripheral oxygen saturation, and end-tidal carbon dioxide) were recorded three times: preinsufflation, 10 minutes after insufflation, and 10 minutes after desufflation. Anesthetic times and surgery times were also all recorded.

ICG elimination tests were done as described by Sakka et al. [14] using the noninvasive LiMON. Each patient received an ICG finger clip that was connected to a liver function monitor. A dose of 0.3 mg/kg ICG (ICGPULSION; Pulsion Medical Systems) was given through a cubital fossa vein as a bolus and immediately flushed with 10 mL of normal saline. ICG-PDR was calculated 5 minutes after induction (ICG-PDR 1) and before extubation (ICG-PDR 2).

Serum aspartate aminotransferase (AST), alanine aminotransferase (ALT), and total bilirubin levels were all recorded preoperatively, 1 hour, and postoperative 24 hours after surgery.

Side effects, such as hypersensitivity, flushing, bronchospasm, and laryngospasm, were also all recorded.

### 2.1. Statistical Analysis

The numeric results were expressed as mean ± SD, and categorical results were expressed as a number. Differences between groups were assessed using Student's *t*-test for age, weight, hemodynamic values, and ICG-PDR. Mann Whitney *U* test for nonnormal distributed data for duration of anesthesia time and surgery time, AST, ALT, and bilirubin. Intragroup comparisons of normally distributed ISG-PDR paired sample *t*-test with Bonferroni correction. Nonnormal distribution of AST, ALT, and bilirubin of the Wilcoxon signed rank test with Bonferroni correction was used for intragroup comparisons. Sex, ASA data were compared using Chi-square test. Statistica 7.0 (StatSoft Inc., Tulsa, OK, USA) statistical software was used for statistical analysis. A *P* value < 0.05 was considered statistically significant.

## 3. Results

Sex, age, weight of patients, duration of anesthesia time, duration of surgery time, and ASA scores are shown in Table 1. There were no statistical differences between groups (*P* > 0.05).

There were no statistical significant difference between groups about heart rate, mean arterial pressure, peripheral oxygen saturation, and end-tidal carbon dioxide (*P* > 0.05) (Table 2).

The ICG-PDR 1 values for Groups I and II were as follows: 26.78 ± 4.2% per min versus 26.01 ± 2.4% per min (*P* > 0.05). ICG-PDR 2 values were found to be 25.63 ± 2.1% per min in Group I versus 19.06 ± 2.2% per min in Group II (*P* < 0.05). There was a statistically significant decrease between baseline and postoperative ICG-PDR values in Group II compared to Group I (*P* < 0.05). These data are shown in Table 3.

The preoperative AST values (normal range 0–34 U/L) were 24.63 ± 8.1 U/L versus 22.01 ± 8.1 U/L in Groups I and II (*P* > 0.05), respectively. The postoperative 1st-hour serum AST level was as follows: 26.78 ± 7.2 U/L versus 34.06 ± 8.2 U/L (*P* < 0.05). Statistically, there was an increase between baseline and postoperative 1st-hour serum AST level in Group II (*P* < 0.05) compared to Group I (Table 4). The postoperative 24th-hour serum AST values were 27.70 ± 8.8 U/L versus 29.20 ± 8.1 U/L (*P* > 0.05) (Table 4).

The preoperative ALT values (normal range 10–49 U/L) were 24.5 ± 7.5 U/L versus 24.91 ± 18.9 U/L in Groups I and II (*P* > 0.05), respectively. The postoperative 1st-hour serum ALT level was as follows: 28.85 ± 10.4 U/L versus 38.52 ± 10.16 U/L (*P* < 0.05). Statistically, there was an increase between baseline and postoperative 1st-hour serum ALT level in Group II (*P* < 0.05) compared to Group I (Table 4). The postoperative 24th-hour serum ALT levels were 30.62 ± 8.3 U/L versus 34.71 ± 14.8 U/L. There was no statistically significant difference between groups (*P* > 0.05) (Table 4).

The preoperative bilirubin values (normal range 0.3–1.3 mg/dL) were 0.67 ± 0.34 mg/dL versus 0.69 ± 0.57 in Groups I and II (*P* > 0.05). The postoperative 1st-hour serum bilirubin activities were as follows: 0.73 ± 0.39 mg/dL versus 0.75 ± 0.62 mg/dL, and the postoperative 24th-hour serum bilirubin activities were 0.71 ± 0.28 mg/dL versus 0.73 ± 0.52 mg/dL. No statistically significant difference was detected at any time (*P* > 0.05) (Table 4).

No side effects were recorded during the study period.

## 4. Discussion

Worldwide, laparoscopic cholecystectomy is most often performed by pumping CO_2_ into the abdominal cavity. To provide good exposure of the surgical field, generally 10 to 15 mmHg pressure ranges are used during pneumoperitoneum [8, 9, 12, 18].

The effects of pneumoperitoneum on splanchnic and hepatic perfusion are not clearly understood. Junghans et al. [19] demonstrated in a pig model that intra-abdominal pressure greater than 12 mmHg may induce a reduction in splanchnic and hepatic perfusion. In this study, the authors used one of three intraperitoneal pressures (8, 12, and 15 mmHg). In another recent study [20], the authors installed IAPs of 7 and 14 mmHg in healthy pigs and demonstrated that higher IAPs decrease portal and superficial hepatic blood flow. They concluded that the derangement in the splanchnic compartment is dependent upon carbon dioxide pneumoperitoneum. Blobner et al. [21] demonstrated that an intra-abdominal pressure of less than 16 mmHg induces an increase in mesenteric artery and portal venous blood flow, due to local vasodilative effects of CO_2_ on splanchnic vessels. The authors found that intra-abdominal pressure of more than 16 mmHg is associated with a decrease in splanchnic perfusion.

Several previous studies reported the effects of pneumoperitoneum on splanchnic and liver perfusion [7, 22]. Meierhenrich et al. [7] demonstrated that induction of CO_2_ pneumoperitoneum with an IAP of 12 mmHg is associated with an increase in hepatic perfusion in healthy adults. The authors do not have a definite explanation for these findings. Another study, done by Sato et al. [22], compared hepatic blood flow and function in patients undergoing laparoscopic cholecystectomy with an intra-abdominal pressure of 9–12 mmHg and concluded that laparoscopic cholecystectomy might impair hepatic function because of the high pressure.

Dynamic liver function tests, such as ICG-PDR, should provide better direct measurement of liver function. Thus, this has been suggested as a marker of global hepato-splanchnic blood flow, and the normal range is 18% to 25% per minute [13, 23, 24]. In our study, like what was done in other recent studies, we used 10 and 14 mmHg insufflation pressures, and we found that the ICG-PDR values decreased in Group II compared to Group I (25.63 ± 2.1% per minute versus 19.06 ± 2.2% per minute) at the end of the operation.

Morino et al. [25] evaluated the effects of pneumoperitoneum on hepatic function in patients treated with laparoscopic procedures. The cholecystectomies were done with pneumoperitoneum at 10 mmHg and at 14 mmHg and found that all patients had a postoperative increase in AST and ALT levels. They suggested that patients with severe hepatic failure should probably not be subjected to prolonged laparoscopic procedures. In another recent study [10], the authors demonstrated statistically significant increase in ALT and AST in the laparoscopic cholecystectomy group at 14 mmHg of CO_2_ pressure. In our study, we found an increase between baseline and postoperative 1st-hour serum AST and ALT levels in Group II compared to Group I. Also, an increase was detected between the preoperative and postoperative 1st-hour serum bilirubin values, but no statistical difference was detected. We believe that the main reason for this was high insufflation pressure.

In conclusion, the results show that 14 mmHg pressure pneumoperitoneum decreased the blood flow to the liver and increased postoperative 1st-hour serum AST and ALT levels. We think that 10 mmHg pressure pneumoperitoneum is superior to 14 mmHg pressure pneumoperitoneum in laparoscopic cholecystectomy.

## Figures and Tables

**Table 1:** Demographic data.

	Group I (*n* = 20)	Group II (*n* = 23)
Sex (M/F)	3/17	14/9
Age (year)	49.40 ± 12.7	51.73 ± 10.5
Weight (kg)	83.8 ± 11.0	84.21 ± 12.1
Anesthesia time (min)	63.5 ± 20.4	68.9 ± 24.3
Surgery time (min)	50.2 ± 19.1	58.5 ± 24.5
ASA (I/II)	12/8	14/9

Data are presented as range (mean ± SD) median unless otherwise indicated. ASA: American Society of Anesthesiologists.

No statistical difference between groups (*P* > 0.05).

**Table 2:** Hemodynamic variables.

		Group I (*n* = 20)	Group II (*n* = 23)
Heart rate (pulse/minute)	Preinsufflation	79.28 ± 15.38	82.00 ± 11.55
	10 min after insufflation	81.52 ± 16.20	76.88 ± 8.29
	10 min after desufflation	78.24 ± 15.86	77.08 ± 10.84

Mean arterial pressure (mmHg)	Preinsufflation	100.13 ± 14.83	98.47 ± 13.52
	10 min after insufflation	105.44 ± 16.68	97.16 ± 16.83
	10 min after desufflation	95.64 ± 15.43	100.08 ± 12.84

Peripheral oxygen saturation (%)	Preinsufflation	99.30 ± 0.73	99.20 ± 0.71
	10 min after insufflation	99.15 ± 1.08	99.10 ± 1.02
	10 min after desufflation	99.10 ± 0.85	99.10 ± 0.71

End-tidal carbon dioxide (mmHg)	Preinsufflation	31.13 ± 7.9	32.00 ± 7.1
	10 min after insufflation	35.17 ± 8.1	35.42 ± 8.3
	10 min after desufflation	31.01 ± 7.1	31.42 ± 7.0

Data are presented as range (mean ± SD) median unless otherwise indicated.

No statistical difference between groups (*P* > 0.05).

**Table 3:** ICG-PDR values.

	Group I (*n* = 20)	Group II (*n* = 23)
ICG-PDR 1	26,78 ± 4.2	26.01 ± 2.4
ICG-PDR 2	25.63 ± 2.1	19.06 ± 2.2^∗€^

ICG-PDR: indocyanine green plasma disappearance rate.

ICG-PDR 1, measurements were made after induction.

ICG-PDR2, measurements were after the end of the operation.

*P < 0.05, ICG-PDR values between groups.

^€^ *P* < 0.05, there was a statistically significant decrease between baseline and postoperative ICG-PDR values in Group II compared to Group I.

**Table 4:** Serum AST (U/L), ALT (U/L), and total bilirubin (mg/dL) values.

	Group I (*n* = 20)	Group II (*n* = 23)
Preoperative AST	24.63 ± 8.1	22.01 ± 8.1
Postoperative 1st-hour AST	26.78 ± 7.2	34.06 ± 8.2^*∗*€^
Postoperative 24th-hour AST	27.70 ± 8.8	29.20 ± 8.1
Preoperative ALT	24.50 ± 7.5	24.91 ± 18.9
Postoperative 1st-hour ALT	28.85 ± 10.4	38.52 ± 10.16^*∗*€^
Postoperative 24th-our ALT	30.62 ± 8.3	34.71 ± 14.8
Preoperative bilirubin	0.67 ± 0.34	0.69 ± 0.57
Postoperative 1st-hour bilirubin	0.73 ± 0.39	0.75 ± 0.62
Postoperative 24th-hour bilirubin	0.71 ± 0.28	0.73 ± 0.52

AST: serum aspartate aminotransferase (normal range 0–34 U/L).

ALT: serum alanine aminotransferase (normal range 10–49 U/L).

Bilirubin values: normal range 0.3–1.3 mg/dL.

*P < 0.05, the postoperative 1st-hour serum AST and ALT level between groups.

^€^ *P* < 0.05, there was an increase between baseline and postoperative 1st-hour serum AST and ALT level in Group II compared to Group I.
